# Transcriptional coactivator with PDZ‐binding motif is required to sustain testicular function on aging

**DOI:** 10.1111/acel.12631

**Published:** 2017-06-14

**Authors:** Mi Gyeong Jeong, Hyuna Song, Ji Hyun Shin, Hana Jeong, Hyo Kyeong Kim, Eun Sook Hwang

**Affiliations:** ^1^ College of Pharmacy and Graduate School of Pharmaceutical Sciences Ewha Womans University Seoul 03760 Korea

**Keywords:** apoptosis, fertility, p53‐p21, senescence, sperm counts, TAZ deficiency

## Abstract

Transcriptional coactivator with PDZ‐binding motif (TAZ) directly interacts with transcription factors and regulates their transcriptional activity. Extensive functional studies have shown that TAZ plays critical regulatory roles in stem cell proliferation, differentiation, and survival and also modulates the development of organs such as the lung, kidney, heart, and bone. Despite the importance of TAZ in stem cell maintenance, TAZ function has not yet been evaluated in spermatogenic stem cells of the male reproductive system. Here, we investigated the expression and functions of TAZ in mouse testis. TAZ was expressed in spermatogenic stem cells; however, its deficiency caused significant structural abnormalities, including atrophied tubules, widened interstitial space, and abnormal Leydig cell expansion, thereby resulting in lowered sperm counts and impaired fertility. Furthermore, TAZ deficiency increased the level of apoptosis and senescence in spermatogenic cells and Leydig cells upon aging. The expression of senescence‐associated β‐galactosidase (SA‐βgal), secretory phenotypes, and cyclin‐dependent kinase inhibitors (p16, p19, and p21) significantly increased in the absence of TAZ. TAZ downregulation in testicular cells further increased SA‐βgal and p21 expression induced by oxidative stress, whereas TAZ overexpression decreased p21 induction and prevented senescence. Mechanistic studies showed that TAZ suppressed DNA‐binding activity of p53 through a direct interaction and thus attenuated p53‐induced p21 gene transcription. Our results suggested that TAZ may suppress apoptosis and premature senescence in spermatogenic cells by inhibiting the p53‐p21 signaling pathway, thus playing important roles in the maintenance and control of reproductive function.

**Abbreviations**
ADM: Adriamycin
SA‐βgal: Senescence‐associated β‐galactosidase
TAZ: Transcriptional co‐activator with PDZ‐binding motif
YAP: Yes‐associated protein

## Introduction

Transcriptional co‐activator with PDZ‐binding motif (TAZ), also referred to as WW domain‐containing transcriptional regulator 1, was first identified as a phosphorylated protein that interacts with 14‐3‐3 proteins in the cytosol and translocates to the nucleus upon dephosphorylation (Kanai *et al*., 2000). TAZ associates with various transcription factors and regulates expression of their target genes during cell differentiation and organ development (Hansen *et al*., 2015). Although the interaction of TAZ with runt‐related transcription factor 2 enhances osteoblast differentiation, its interaction with peroxisome proliferator‐activated receptor γ decreases adipocyte differentiation (Cui *et al*., 2003; Hong *et al*., 2005). TAZ has also been shown to interact with the myogenic regulatory factor, MyoD, and accelerates MyoD‐induced myogenic differentiation and muscle regeneration (Jeong *et al*., 2010). In addition, TAZ plays key roles in regulating organogenesis of the heart, lung, thyroid, and neural crest by regulating the expression of T‐box transcription factor 5, thyroid transcription factor‐1, paired box homeotic gene 3, and members of transcriptional enhancer activator domain family (Park *et al*., 2004; Mahoney *et al*., 2005; Murakami *et al*., 2005; Zhang *et al*., 2009). TAZ also plays a role in cancer development because it is a key mediator of Hippo signaling. Hippo signaling suppresses TAZ activity through induction of TAZ phosphorylation and degradation, whereas the loss of Hippo signaling elevated TAZ activity in cancer cells, resulting in uncontrolled cell proliferation (Moroishi *et al*., 2015). TAZ is thus suggested to have an oncogenic activity in human cancer such as nonsmall cell lung cancer (Zhou *et al*., 2011; Noguchi *et al*., 2014).

The *in vivo* physiological functions of TAZ have been identified using TAZ knockout (KO) mice. Conventional TAZ KO mice showed alveolarization defects and pulmonary emphysematous changes in the lung (Makita *et al*., 2008). This was partly explained by the inhibitory activity of TAZ against lung‐specific thyroid transcription factor‐1 function (Park *et al*., 2004; Mitani *et al*., 2009). TAZ KO mice also developed severe kidney defects causing symptoms resembling polycystic kidney disease in humans (Hossain *et al*., 2007; Makita *et al*., 2008). Consistently, TAZ deletion in the cap mesenchyme of the developing kidney resulted in abnormal nephron formation and large cysts (Reginensi *et al*., 2013). TAZ deficiency resulted in polycystin 2 accumulation in the kidney via loss of multiprotein E3 ubiquitin ligase activity and hyperactivation of Wnt/β‐catenin signaling (Tian *et al*., 2007; Varelas *et al*., 2010). Thus, it suggested that TAZ may suppress the development of polycystic kidney disease. While TAZ functioned as a coactivator of T‐box transcription factor 5 in cardiac and limb development (Murakami *et al*., 2005), TAZ deletion did not produce any severe heart defects (Xin *et al*., 2013). However, the loss of both TAZ and Yes‐associated protein (YAP), a TAZ paralog, caused lethal cardiomyopathy by reducing cardiomyocyte proliferation in a Hippo/Yap signaling‐dependent manner (Xin *et al*., 2013; Lin & Pu, 2014). More recently, cranial neural crest‐specific deletion of both YAP and TAZ was reported to cause cerebellar aplasia and hydrocephalus, indicating important roles for TAZ in neuronal differentiation (Ahmed *et al*., 2015; Wang *et al*., 2016).

Despite this wealth of information on TAZ functions in many types of tissues or organs, its functions in the reproductive organs have not yet been elucidated. In this study, we confirmed that TAZ is expressed in mouse testis containing abundant germinal stem cells and characterized novel TAZ functions in the reproductive organ.

## Results

### TAZ deficiency caused structural abnormality and functional defects in the testis

**Figure 1:**
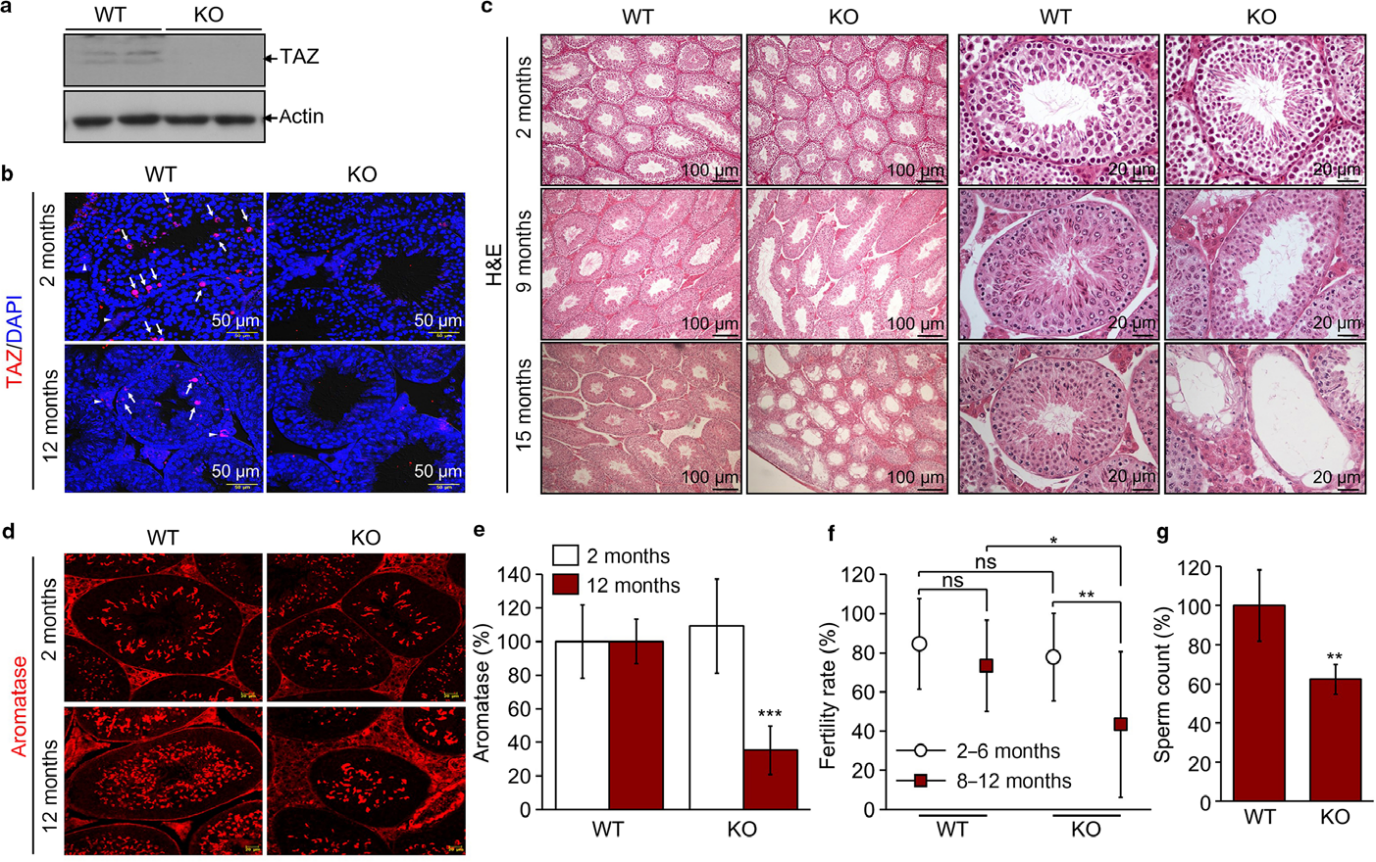
Abnormal morphology and functional defects of TAZ‐deficient testis. (a) Immunoblotting images using TAZ and actin antibodies to testis protein lysate. (b) Immunohistochemical analysis of WT and KO (2 and 12 months old, *n* = 6/group) testis tissues using TAZ antibody (red). Spermatogonial and interstitial Leydig cells are marked by arrows and arrowheads, respectively. Nuclei were counterstained with DAPI (blue). (c) H&E staining and microscopic observation of testis tissue sections of WT and KO mice (2, 9, and 12 months old, *n* = 6/group). (d) Immunofluorescence staining of aromatase (red) in WT and KO testis (2 and 12 months old, *n* = 6/group). (e) Quantitative analysis of aromatase‐positive spermatogenic cells in seminiferous tubules (*n* = 6/group). (f) Male fertility rate of WT and KO mice (2–6 months and 8–12 months old, *n* = 15/group) from matings with WT females (*n* = 45/group). (g) Sperm counts from testis of WT and KO mice (8–12 months old, *n* = 10/group). Representative images from three independent experiments are shown in panel a–d. The data in panel e–g indicate the mean ± SD of three independent experiments. *P < 0.05, **P < 0.005, ***P < 0.0005.

To examine the roles of TAZ in the male reproductive system, we first demonstrated that TAZ was expressed in the testis, particularly in the spermatogenic cells of the seminiferous tubules and interstitial Leydig cells, as evidenced by immunoblotting and immunohistochemistry (Fig. 1a,b). Further structural examination by H&E staining confirmed that TAZ was essential for the structural maintenance because a TAZ deficiency resulted in atrophied tubules and widened interstitial spaces. Age‐related atrophied tubules and abnormal expansion of the Leydig cells were enhanced in TAZ KO testis compared to WT testes (Fig. 1c). We next assessed spermatogenesis in TAZ KO mice by analyzing the expression of aromatase, which is essential for spermatogenesis and is highly expressed in round spermatids and spermatozoa (Robertson *et al*., 1999). Compared to WT testicles, those from KO mice revealed decreased aromatase expression with aging (Fig. 1d). Further quantitative analysis also confirmed a significant reduction of aromatase‐positive spermatogenic cells in aged KO testes (Fig. 1e). While young WT and KO males expressed similar fertility rates, aged KO males exhibited significant decreases in fertility and sperm counts (Fig. 1f,g).

### Increased apoptosis in TAZ‐deficient testes

**Figure 2:**
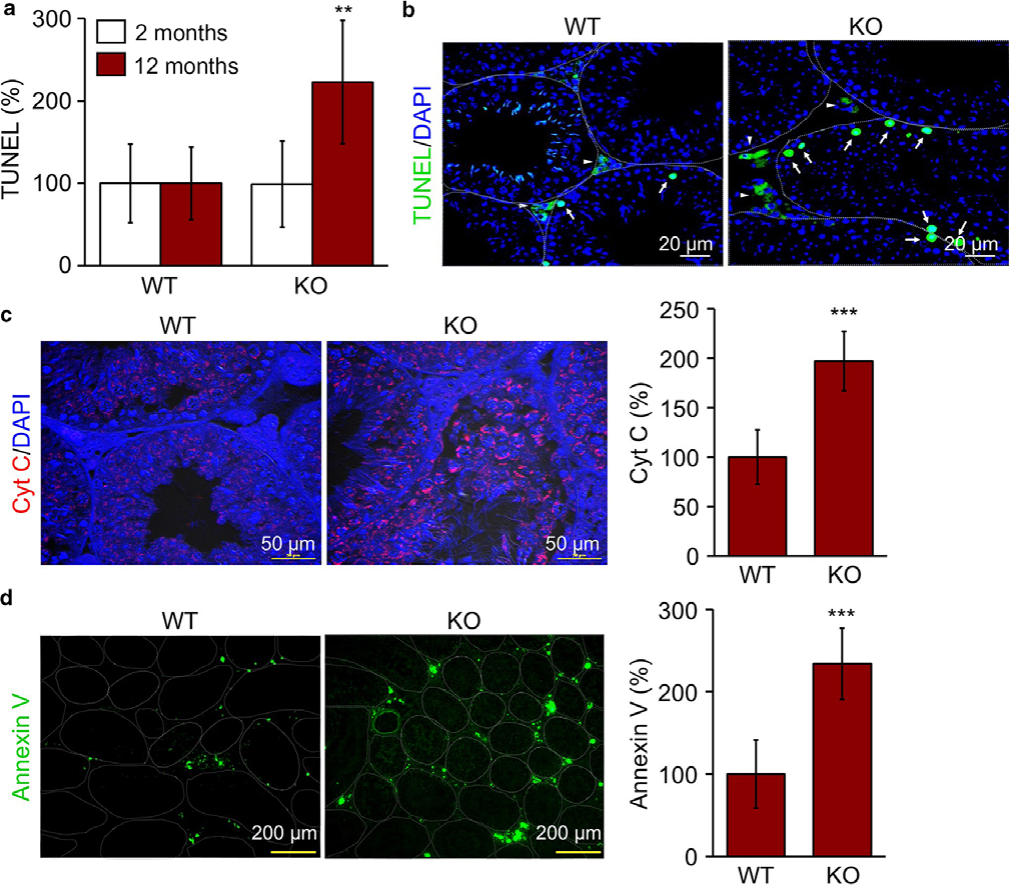
Increased apoptosis in TAZ KO testicles. Testis tissue sections (*n* = 6 per group) obtained from 2‐ and 12‐month‐old WT and KO mice were analyzed by immunohistochemistry. (a) TUNEL‐positive cells were counted at 10 different images of each mouse testis and expressed as a percentage of WT value. (b) Representative images of TUNEL‐positive cells (green). (c) Immunohistochemistry with antibody against cytochrome C (red) and quantitative analysis of Cyt C‐positive cells by ImageJ program. Nuclei were counterstained with DAPI (blue) in b and c. (d) Representative images of Annexin V staining and quantitative analysis of Annexin V‐expressing cells (green). The data are expressed as the mean ± SD of 10 different regions from six different samples. **P < 0.005, ***P < 0.0005.

As TAZ‐deficient testes showed tubule atrophy and decreased sperm counts, we evaluated whether spermatogenic cell survival was affected by TAZ deficiency. Interestingly, TUNEL staining to detect apoptotic cells revealed that the number of TUNEL‐positive apoptotic cells increased in TAZ KO testes from 12‐month‐old mice (Fig. 2a). Apoptotic cell death of spermatogonial cells and interstitial Leydig cells was prominently observed (Fig. 2b). Cytochrome C staining and quantitative analysis confirmed that spermatogenic cells preferably underwent apoptosis under TAZ‐deficient conditions with increasing age (Fig. 2c). In addition, Annexin V staining, which preferentially identifies the damaged cells in testicles (Koji *et al*., 2001), showed significantly increased apoptosis in testicles from aged TAZ KO mice (Fig. 2d). Annexin V‐positive cells were found in the interstitial space of testis and more prominently in aged TAZ KO testes than in WT testes (Fig. 2d).

### Senescence‐associated phenotypes are prominent in TAZ‐deficient testes

**Figure 3:**
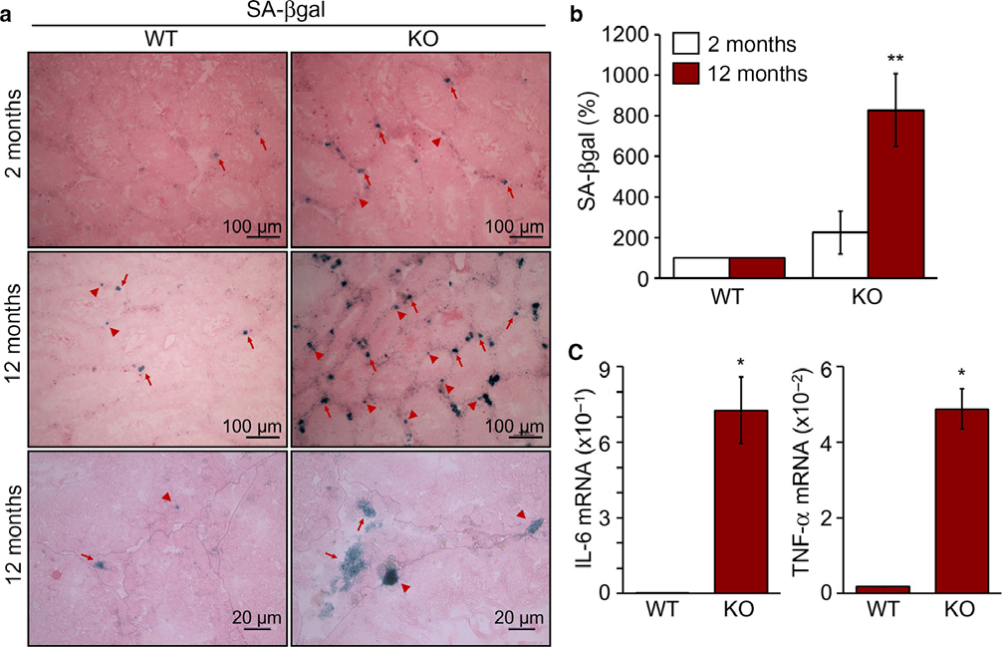
Enhanced senescence phenotypes by TAZ deficiency. (a) SA‐βgal staining of frozen testis sections of 2‐ and 12‐month‐old WT and KO mice (*n* = 6/group), followed by observation with a microscope. Arrows and arrowheads indicate SA‐βgal‐positive spermatogonial and Leydig cells, respectively. (b) Quantitative analysis of SA‐βgal expression in WT and KO. **P < 0.005. (c) Total RNA was extracted from the testis of WT and KO mice (12 months old, *n* = 6). Relative transcript level of IL‐6 and TNF‐α determined by a quantitative real‐time PCR analysis. *P < 0.05.

Because TAZ KO mice showed abnormal testicular structure, defective reproductive function, and increased spermatogenic cell loss, which are signature features of testicular aging, we examined age‐related testicular phenotypes in TAZ KO mice. Interestingly, analyzing SA‐βgal activity as a marker of cellular senescence confirmed that testicular senescence had significantly increased in TAZ KO mice at both 2 and 12 months of age, and this effect was more enhanced with increasing age (Fig. 3a). In particular, spermatogonial cells and interstitial Leydig cells were positively stained with SA‐βgal (Fig. 3a). TAZ deficiency significantly accelerated testicular senescence, as evidenced by a quantitative analysis of SA‐βgal activity (Fig. 3b). Moreover, the senescence‐associated secretory proteins, IL‐6 and TNF‐α, were significantly upregulated in aged TAZ KO testis, compared to their expression levels in WT testis (Fig. 3c).

### DNA damage and senescence responses are enhanced by TAZ deficiency

**Figure 4:**
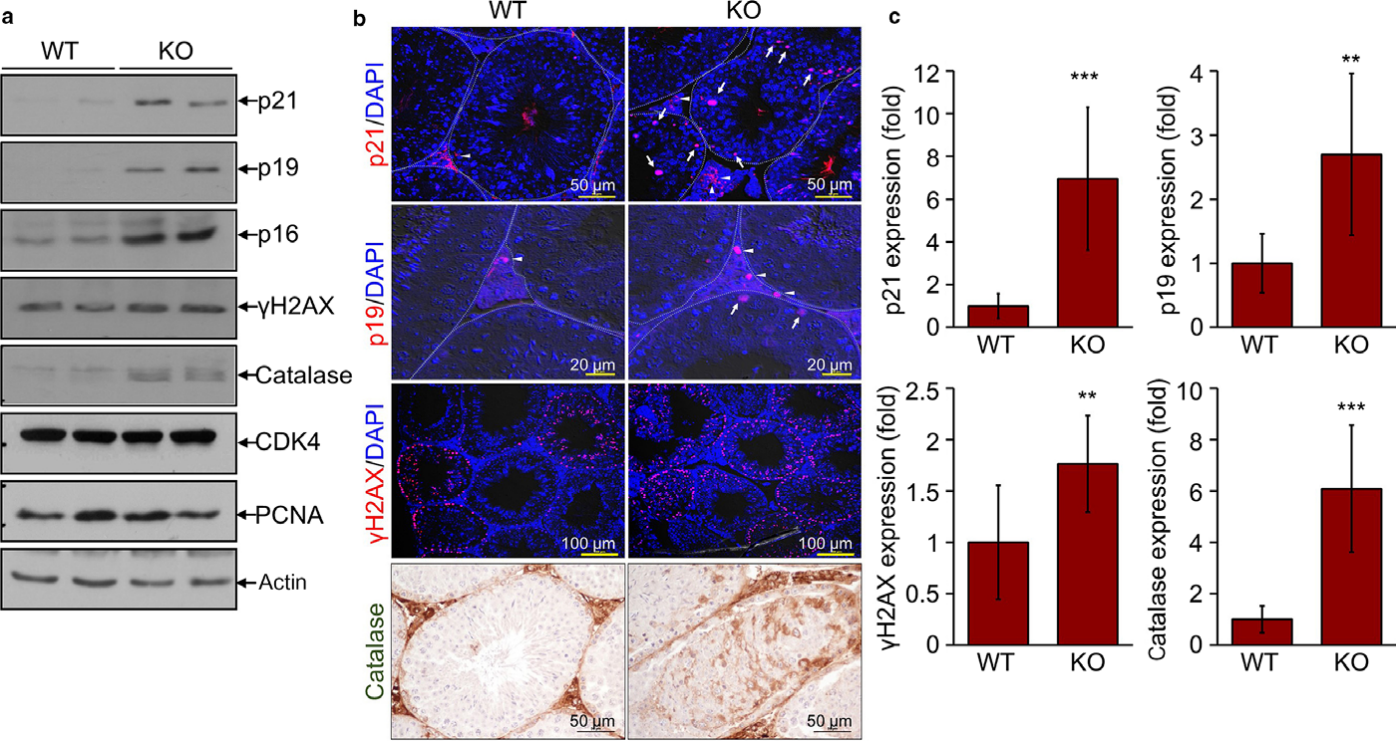
Increased level of senescence and damage markers in TAZ KO testicles. Testes were collected from WT and KO mice (12 months old, *n* = 6/group). (a) Immunoblotting analysis with antibodies against p21, p19, p16, γH2AX, catalase, CDK4, PCNA, and actin. (b) Testis tissue sections were analyzed by immunohistochemistry using antibodies against p21, p19, γH2AX, and catalase. Representative images are shown. Arrows and arrowheads indicate positive cells in spermatogonial and the interstitial Leydig cells, respectively. (c) Quantitative image analysis of p21, p19, γH2AX, and catalase from 10 images of six mice using ImageJ software (c). **P < 0.005, ***P < 0.0005.

We further examined the effects of TAZ on the expression of protein biomarkers associated with cell cycle arrest and senescence. TAZ deficiency caused upregulation of the cyclin‐dependent kinase inhibitors p21, p19, and p16, while expression of cyclin‐dependent kinase 4 (CDK4) and proliferating cell nuclear antigen (PCNA) was comparable between the WT and KO groups. Damage‐related γH2AX and catalase were induced in the testicles of TAZ KO mice (Fig. 4a). Immunohistochemical analysis revealed that the cell cycle inhibitors p21 and p19 were upregulated in spermatogonial and interstitial Leydig cells of TAZ KO mice (Fig. 4b). We also found that expression of γH2AX and catalase was augmented in the seminiferous tubules of TAZ KO testis by microscopic observations (Fig. 4b) and a quantitative analysis (Fig. 4c).

### TAZ directly modulates stress‐induced cellular senescence

**Figure 5:**
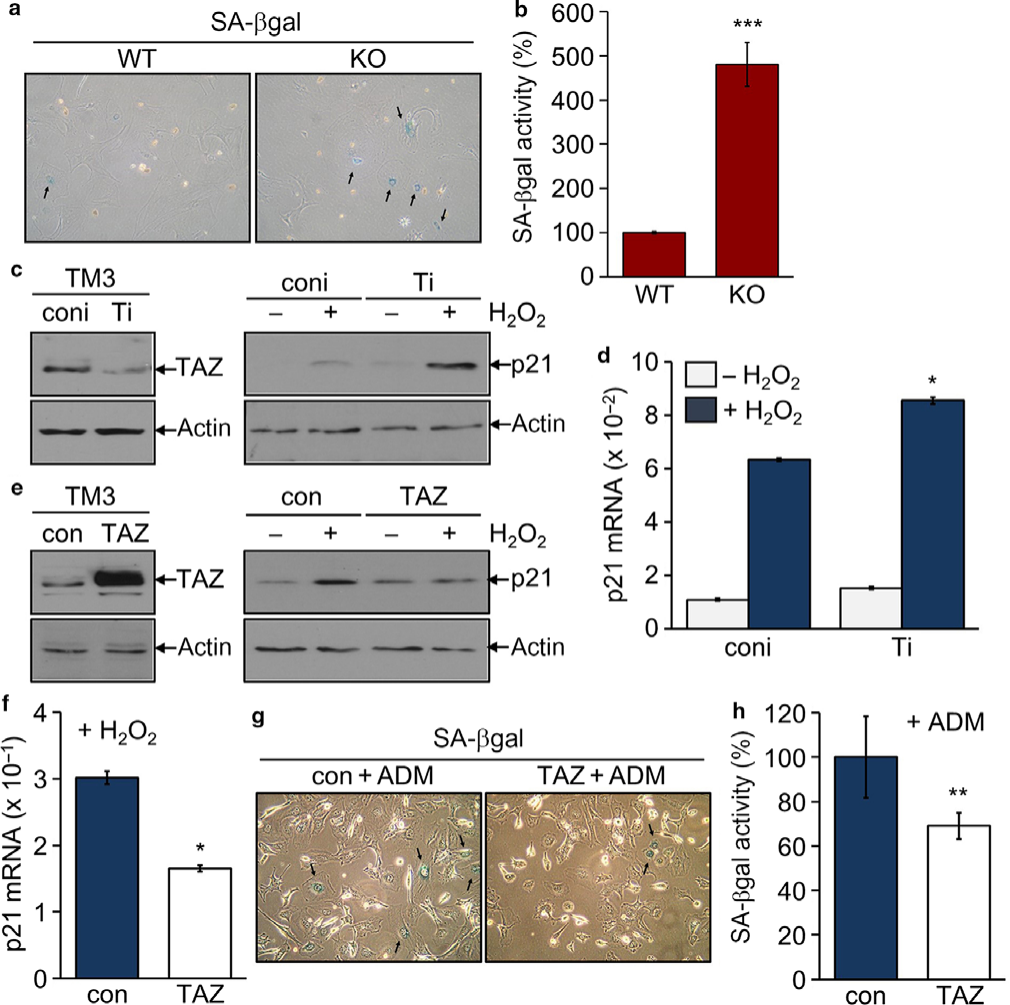
Suppression of p21 expression and senescence by TAZ. (a, b) Primary testicular cells isolated WT and TAZ KO mice (12 months old, *n* = 6 each group) were maintained for three passages. Cells were subjected to a SA‐βgal staining (a) and β‐galactosidase activity assay (b). Data are mean ± SD of six experiments. ***P < 0.0005. (c–h) Stable cell lines were established in mouse TM3 cells: knock‐down control (coni), TAZ knock‐down (Ti), control (con), and TAZ stable cells (TAZ), and treated with 100 μM H_2_O_2_ for 4 h. Immunoblotting analysis of TAZ, p21, and actin (c, e). Quantitative analysis of p21 mRNA level (d, f). *P < 0.05. Stable cells, con and TAZ, were incubated with 50 nM ADM for 3 days and assayed with a SA‐βgal staining (g) and β‐galactosidase activity assay (h). **P < 0.005.

As TAZ deficiency increased testicular senescence *in vivo* with age, we attempted to investigate whether TAZ directly affected cellular senescence *in vitro* in cell culture system. Primary testicular cells isolated from WT and TAZ KO mice were maintained under serum‐free conditions to induce cellular senescence. The SA‐βgal staining increased in KO cells compared to WT cells (Fig. 5a). Quantitative evaluation of SA‐βgal activity revealed a 500% increase in KO cells (Fig. 5b). Furthermore, TAZ knock‐down in the mouse Leydig TM3 cells further increased oxidative stress‐induced p21 protein and mRNA expression (Fig. 5c,d). In contrast, TAZ overexpression in TM3 cells inhibited oxidative stress‐induced p21 expression (Fig. 5e,f). Consistently, cells stably overexpressing TAZ showed decreased ADM‐induced SA‐βgal positivity, while control cells underwent senescence after treatment with ADM (Fig. 5g,h).

### TAZ suppresses the transcriptional and DNA‐binding activity of p53 through a direct interaction

**Figure 6:**
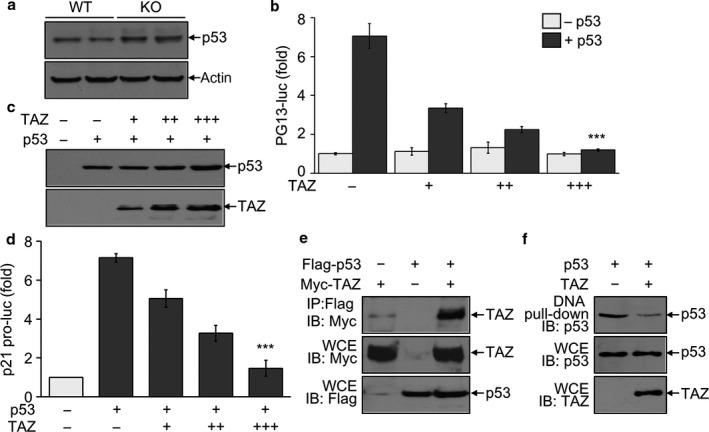
Suppression of p53 activity by TAZ via direct interaction. (a) Immunoblotting analysis of p53 in the testis of WT and KO mice (12 months old, *n* = 6/group). (b–d) 293T cells were transfected with the reporter gene either PG13‐luc (b) or p21‐luc (d) and/or TAZ expression vector. Expression of p53 and TAZ was detected by immunoblotting analysis (c). The reporter activity was determined after normalization with β‐galactosidase activity and given as the fold induction compared to mock control. (e, f) 293T cells were transfected with Flag‐tagged p53 and Myc‐tagged TAZ expression vector. Whole cell extracts (WCE) were harvested and used for immunoprecipitation with anti‐Flag antibody, followed by Immunoblot analysis (e). Or cell extracts were incubated with biotinylated p21 promoter, followed by precipitation and immunoblotting (f). The data in panel b and d indicate the mean ± SD of three independent experiments. ***P < 0.0005. Representative blots of at least three experiments are shown in panels a, c, e, and f.

Transcription of p21 gene is tightly regulated by the expression level and activity of p53 (Campisi, 2013; Munoz‐Espin & Serrano, 2014). Because p21 expression was significantly increased in TAZ‐deficient testes, we examined the effect of TAZ on the expression and activity of p53. The expression level of testicular p53 was not affected by TAZ deficiency (Fig. 6a). However, p53‐induced reporter activity was suppressed by TAZ expression in a dose‐dependent manner, while the p53 expression level was comparable regardless of TAZ overexpression (Fig. 6b,c). Consistently, TAZ dose‐dependently suppressed the p21 promoter activity that was increased by p53 overexpression (Fig. 6d). We next showed that TAZ directly associated with p53 by protein–protein interaction assay (Fig. 6e). This interaction between TAZ and p53 resulted in attenuated DNA‐binding activity of p53, as evidenced by DNA pull‐down assay (Fig. 6f).

## Discussion

In the present study, we first assessed the expression of TAZ in spermatogenic cells and interstitial Leydig cells in mouse testis and found that a TAZ deficiency promoted the age‐related loss of testicular functions, likely as a result of significant increases in apoptosis and cellular senescence. TAZ deficiency increased expression of the senescence‐associated proteins p21, p19, and p16, whereas TAZ inhibited p53‐dependent p21 expression and the subsequent development of senescence. Our results provided the first demonstration that TAZ was crucial for maintaining testicular structure and functions.

The birthrate of TAZ KO mice is approximately 5%, and KO mice are rarely viable at 4 weeks of age because of kidney and lung failure (Hossain *et al*., 2007). While attempting to obtain a sufficient number of TAZ KO mice by breeding KO males with heterozygote females, we observed that KO mice were less fertile than WT mice or heterozygotes and that a small fraction of KO mice survived until the ages of 12–15 months, despite their defects. We found that TAZ deficiency caused structural abnormality with increasing age and also reduced fertility and fecundity. Male fertility and fecundity are generally evaluated in terms of sperm concentration, motility, and morphology and are also validated based on the pregnancy rate in mated females. Although aromatase‐positive spermatogenic cells and sperm counts decreased in the testes of TAZ KO males, it remains yet to be clarified whether the sperm motility and morphology are affected by TAZ deficiency. Mature sperm cells (spermatozoa) are continuously produced from spermatogonial stem cells through proliferation and differentiation into spermatocytes and spermatids in the seminiferous tubules (de Rooij, 2001). Spermatogenesis is initiated by testosterone production in interstitial Leydig cells (Russell *et al*., 1987) and is tightly regulated in germ cells by transcription factors, such as heat shock factor 2 and *ovo* homolog‐like 1 in germ cells (Bettegowda & Wilkinson, 2010). TAZ may directly modulate the survival of spermatogenic cells including spermatogonial cells, spermatocytes, and spermatozoa, and it may also regulate spermatogenesis by controlling hormonal homeostatic regulation (de Rooij, 2001). Despite the importance of our findings, severe inflammation in the lung and kidney of TAZ KO mice may cause defective spermatogenesis and reproductive failure. Thus, generation of testis‐specific TAZ conditional KO mice can be valuable to identify novel TAZ functions in the male reproduction system, including spermatogenic cells, sertoli cells, and Leydig cells. It will also provide advanced knowledge regarding the cellular and molecular mechanisms underlying the protective role of TAZ in the testes.

In addition to the novel function of TAZ in the regulation of reproductive activity, we found that TAZ deficiency promoted cellular senescence and further increased age‐associated disorders. Cellular senescence is an irreversible arrest of cell proliferation caused by aging or pathological stimuli via the upregulation of cell cycle inhibitors and tumor suppressors, including p53/p21 and p16/pRB (Campisi, 2013; Munoz‐Espin & Serrano, 2014). Here, we found that TAZ deficiency increased SA‐βgal activity and promoted p21 expression in spermatogonial and interstitial Leydig cells. TAZ overexpression inhibited p53‐induced p21 expression through a direct interaction with p53 and partly suppressed cellular senescence, suggesting that TAZ functions in conjunction with p53‐p21 pathway inhibition in controlling cellular senescence. Although TAZ overexpression prevented testicular cells from undergoing senescence *in vitro*, it will be critical to determine whether TAZ restoration can reverse testicular senescence and functional defects *in vivo*. In addition, hormonal dysregulation in interstitial Leydig cells also causes immature sperm production and male reproductive dysfunction, resulting in Leydig cell dysplasia and testicular senescence (Robertson *et al*., 1999; Hess, 2003). It will also be important to identify more specific and detailed functions of TAZ and its underlying mechanisms in hormone synthesis and homeostatic regulation by interstitial Leydig cells in the testis. Elucidation of the detailed molecular mechanisms underlying TAZ‐mediated regulation of age‐dependent testicular function would provide valuable therapeutic targets and promote the development of novel drugs for treating defective male reproductive function.

## Experimental procedures

### Animals

Male WT C57BL/6 mice purchased from the Jackson Laboratory (Minneapolis, MN, USA) and TAZ KO mice were generated as previously reported (Hossain *et al*., 2007). All mice were bred and maintained in specific pathogen‐free animal facility at Ewha Womans University. All animals were housed under controlled conditions (temperature 22 ± 2 °C, humidity 60 ± 5%, and lighting 12 h light/12 h dark), and all experiments with animals were approved by IACUC (2012‐01‐020 and 15‐006) at Ewha Womans University and conducted by the IACUC guidelines.

### Mating assay

Male WT and TAZ KO mice (2–6 months old, *n* = 15; 8–12 months old, *n* = 15) that had been individually housed were mated with two or three females (10–12 weeks). Vaginal plugs were counted in mated females (*n* = 45 per mating group) every morning after pairing. Each mouse with plug was isolated to a separate cage and carefully observed for pregnancy and delivery. The fertility rates of WT and KO males were determined and expressed as mean ± SD.

### Sperm counts

The sperm counting was performed using 10 aged male WT and TAZ KO (8–12 months old, *n* = 10) mice according to the previous report (Tayama *et al*., 2006). In brief, the testis was separated, minced with small scissors in DMEM, and compressed gently using the plunger of 1‐mL syringe. The sperm suspensions were incubated for 30 min and used for sperm counts using a hemocytometer.

### Hematoxylin and eosin (H&E) staining

Testes obtained from TAZ WT and KO mice at different ages (2–15 months old) were fixed in fresh Bouin's fixative (Sigma‐Aldrich, St. Louis, MO, USA) for 16 h, washed with 70% ethanol, dehydrated, and embedded in paraffin. Tissues were sectioned at a thickness of 5 μm, deparaffinized, rehydrated, and stained with hematoxylin and eosin (Sigma‐Aldrich). Sections were analyzed using a Nikon Eclipse E200 microscope (Nikon, Melville, NY, USA) with attached digital camera (Olympus, Melville, NY, USA).

### TUNEL staining

Testis tissues were obtained from 12‐month‐old WT and KO mice and fixed in 4% paraformaldehyde solution. Tissue sections were subjected to permeabilization with proteinase K solution, incubation with the TdT enzyme and fluorescence‐labeled dUTP (Sigma‐Aldrich), and washing with phosphate‐buffered saline according to the manufacturer's instruction (Sigma‐Aldrich). Slides were analyzed using a fluorescence microscope LSM‐510 META (Carl Zeiss, Gottingen, Germany), and the fluorescence‐positive cells were quantitatively analyzed with ImageJ program.

### Immunohistochemistry

Testis tissues were isolated and were immediately frozen or fixed in 10% neutralized formalin. Paraffin‐embedded tissue sections were deparaffinized, rehydrated, incubated in 1% hydrochloric acid, and washed with distilled water. Frozen sections were fixed in precooled acetone and incubated with 0.3% hydrogen peroxide solution. Tissue slides were blocked and incubated with the primary antibody against Annexin V, cytochrome C, p21, p19, γH2AX, catalase (Abcam, Cambridge, MA, USA), or TAZ (#2149; Cell Signaling Technology, Danvers, MA, USA), and subsequently with the secondary antibody (Molecular Probes, Eugene, OR). Slides were counterstained with 4, 6‐diamidino‐2‐phenylindole (DAPI; 1.5 mg mL^−1^) and quantitatively analyzed using a fluorescence microscope and imagej software.

### Senescence‐associated β‐galactosidase (SA‐βgal) staining

Testes isolated from WT and KO mice were immediately placed on the mold filled with sufficient OCT (Sigma‐Aldrich) and then frozen in liquid nitrogen. Frozen tissues were cut using a frozen section Cryostat (Thermo Fisher Scientific, Waltham, MA, USA) and fixed for 10 min. Slides were rinsed with PBS and stained with β‐galactosidase staining solution (Cell Signaling Technology) overnight. Slides were counterstained with eosin, followed by microscopic observation. Signals on the images were quantitatively analyzed using the imagej software. (free download from National Institutes of Health, Bethesda, MD, USA)

### Cell culture

The mouse Leydig TM3 cell line was purchased from ATCC (CRL‐1714, Manassas, VA, USA) and maintained in DMEM with 10% FBS (Thermo Fisher Scientific). Stable cell lines were established by viral transduction with knock‐down control vector (coni), TAZ knock‐down vector (Ti), TAZ expression vector (T), or mock (con). Stable cell clones were treated with hydrogen peroxide (H_2_O_2_, Sigma‐Aldrich, 100 μm) for 4 h or adriamycin (ADM, Sigma‐Aldrich, 50 nm) for 3 days to induce the cellular senescence. Primary testicular cells were obtained from WT and KO mice and cultured in complete DMEM as reported (Chang *et al*., 2011).

### Reverse transcription and real‐time polymerase chain reaction (PCR)

Total RNA was isolated from testes or mouse Leydig TM3 cells using a TRIzol reagent and reversely transcribed using a cDNA synthesis kit (Invitrogen, Carlsbad, CA, USA). The cDNA is then used as template for real‐time PCR using SYBR Green premix buffer (Thermo Fisher Scientific) and a specific primer set. Primers were as follows: 5′‐cgagaacggtggaactttgac‐3′, 5′‐tcccagacgaagttgccct‐3′ for p21; 5′‐catcttctcaaattcgagtgacaa‐3′, 5′‐tgggagtagacaaggtacaaccc‐3′ for TNF‐α; 5′‐gaggataccactcccaacaga‐3′, 5′‐aagtgcatcatcgttgttcataca‐3′ for IL‐6; and 5′‐agagggaaatcgtgcgtgac‐3′, 5′‐caatagtgatgacctggccgt‐3′ for β‐actin. Real‐time PCR was performed using an ABI‐Prism 7700 sequence detector (Applied Biosystems), and the relative expression level was calculated after normalization to the level of β‐actin.

### Protein–protein interaction assay

Highly transfectable 293T cells were transiently transfected with Flag‐tagged p53 and/or Myc‐tagged TAZ expression vector using calcium phosphate transfection method. Whole cell lysates were harvested and incubated with Flag‐M2 agarose beads (Sigma‐Aldrich), followed by washing. Whole cell lysates and the immune complexes were resolved by SDS‐PAGE and subjected to immunoblotting. Antibody against Flag (Sigma‐Aldrich) or Myc peptide (9E10; Santa Cruz Biotechnology, Santa Cruz, CA, USA) was used for the detection.

### Reporter gene assay

293T cells were transfected with PG13‐luc, a reporter gene containing the multiple copies of p53 consensus binding element linked to the luciferase gene with p53 and/or TAZ expression vector. Or cells were transfected with the p21‐promoter reporter gene (p21 pro‐luc) with p53 and/or TAZ expression vector. A pCMVβ vector was used as an internal control to compensate for the transfection efficiency. Reporter activity was assayed with a luciferase assay kit (Promega, Madison, WI, USA) or a β‐galactosidase assay kit (Thermo Fisher Scientific). Relative luciferase activity was determined after normalization to the β‐galactosidase activity and expressed as a fold induction.

### Protein‐DNA binding assay

293T cells were transfected with p53 with or without TAZ expression vector. Whole cell lysates were extracted using HKMG buffer including protease inhibitors and incubated with biotinylated p53‐response element of the p21 gene promoter (Integrated DNA Technologies, Coralville, IA, USA), followed by the subsequent incubation with streptavidin‐agarose bead (Thermo Fisher Scientific). Whole cell lysates and the protein‐DNA precipitates were then subjected to SDS‐PAGE and immunoblotting with p53 antibody. The p53‐response element of the p21 gene promoter was prepared by annealing two synthetic oligomers: 5′‐TTCAGGAACATGTCTTGACATGTTC‐AGCCCTGGA‐3′; and 5′‐TTCCAGGGCTGAACATGTC‐AAGACATGTTCCTGA‐3′.

### Statistical analysis

All data are expressed as mean ± SD. Results were obtained from at least three independent experiments and analyzed by the one‐way analysis of variance or two‐tailed Student's *t*‐tests. *P* values under 0.05 were considered statistically significant.

## Funding

This work was supported by Mid‐career Research Program of the National Research Foundation of Korea (NRF‐2013R1A2A2A01068302).

## Author contributions

MGJ and HS performed histological analysis. JHS and HJ performed *in vitro* assay including protein interaction and reporter assay. HKK analyzed the data and calculated statistical analysis. ESH designed the experimental plans, analyzed the data, and prepared for the manuscript for publication.

## Conflict of interest

The authors declare no conflict of interest.
